# Cytotoxic Activity and Chemical Composition of the Root Extract from the Mexican Species *Linum scabrellum*: Mechanism of Action of the Active Compound 6-Methoxypodophyllotoxin

**DOI:** 10.1155/2015/298463

**Published:** 2015-07-12

**Authors:** Ivonne Alejandre-García, Laura Álvarez, Alexandre Cardoso-Taketa, Leticia González-Maya, Mayra Antúnez, Enrique Salas-Vidal, J. Fernando Díaz, Silvia Marquina-Bahena, María Luisa Villarreal

**Affiliations:** ^1^Centro de Investigación en Biotecnología, Universidad Autónoma del Estado de Morelos, Avenida Universidad 1001, Colonia Chamilpa, 62209 Cuernavaca, MOR, Mexico; ^2^Centro de Investigaciones Químicas IICBA, Universidad Autónoma del Estado de Morelos, Avenida Universidad 1001, Colonia Chamilpa, 62209 Cuernavaca, MOR, Mexico; ^3^Facultad de Farmacia, Universidad Autónoma del Estado de Morelos, Avenida Universidad 1001, Colonia Chamilpa, 62209 Cuernavaca, MOR, Mexico; ^4^Instituto de Biotecnología, Universidad Nacional Autónoma de México, 62209 Cuernavaca, MOR, Mexico; ^5^Centro de Investigaciones Biológicas, Madrid 28040, Spain

## Abstract

The cytotoxic activity and the chemical composition of the dichloromethane/methanol root extract of *Linum scabrellum* Planchon (Linaceae) were analyzed. Using NMR spectra and mass spectrometry analyses of the extract we identified eight main constituents: oleic acid (**1**), octadecenoic acid (**2**), stigmasterol (**3**), *α*-amyrin (**4**), pinoresinol (**5**), 6 methoxypodophyllotoxin (**6**), coniferin (**7**), and 6-methoxypodophyllotoxin-7-*O*-*β*-D-glucopyranoside (**8**). By using the sulforhodamine B assay, an important cytotoxic activity against four human cancer cell lines, HF6 colon (IC_50_ = 0.57 *μ*g/mL), MCF7 breast (IC_50_ = 0.56 *μ*g/mL), PC3 prostate (IC_50_ = 1.60 *μ*g/mL), and SiHa cervical (IC_50_ = 1.54 *μ*g/mL), as well as toward the normal fibroblasts line HFS-30 IC_50_ = 1.02 *μ*g/mL was demonstrated. Compound **6** (6-methoxypodophyllotoxin) was responsible for the cytotoxic activity exhibiting an IC_50_ value range of 0.0632 to 2.7433 *µ*g/mL against the tested cell lines. Cell cycle studies with compound **6** exhibited a cell arrest in G2/M of the prostate PC3 cancer cell line. Microtubule disruption studies demonstrated that compound **6** inhibited the polymerization of tubulin through its binding to the colchicine site (binding constant *K*_*b*_ = 7.6 × 10^6^ M^−1^). A dose-response apoptotic effect was also observed. This work constitutes the first investigation reporting the chemical composition of *L. scabrellum* and the first study determining the mechanism of action of compound **6**.

## 1. Introduction

The genus* Linum* of the Linaceae family includes more than 200 globally distributed species. The phytochemical profiles that have been reported for the species of* Linum* suggest the presence of some lignan-type secondary metabolites [1]. These kinds of compounds show several important pharmacological activities such as transcriptase reverse inhibition and HIV activity, effects on cardiovascular system, antileishmaniasis properties, hypoglycemic activities, 5-lipoxygenase inhibition, and antifungal, antirheumatic, antipsoriasis, antimalarial, and antiasthmatic properties [2–14]. However, cytotoxic and antitumoral activities are a major interest on these types of lignans [15]. Podophyllotoxin (PTOX) is the most important aryltetralin-lignan for human health in relation to anticancer and antiviral activities. It is used for the treatment of genital warts (condyloma acuminata) caused by the human papilloma virus [15] and for the semisynthesis of the two important antineoplastic agents etoposide and teniposide, which are actually used in the treatment of several cancers, among others: acute myeloid leukemia, Hodgkin's disease, non-Hodgkin's lymphoma, lung cancer (both small cell and non-small cell), gastric cancer, breast cancer, and ovarian cancer [16].

Some species of* Linum* as* L. persicum* and* L. nodiflorum* accumulate PTOX [17], while others as* L. flavum*,* L. mucronatum* ssp.* armenum*,* L. capitatum*,* L. arboretum*,* L. campanulatum*,* L. elegans*,* L. pamphylicum*,* L. tauricum*,* L. thracicum*,* L. sibiricum*, and* L. nodiflorum* accumulate 6-methoxypodophyllotoxin (6MPTOX) [18]. The mechanism of action of PTOX is based on the inhibition of tubulin polymerization through interaction with the binding site of colchicine [19] as well as on the arrest of the cell cycle in metaphase [20–22]. Although the compound 6MPTOX has been reported with cytotoxic activity against murine Ehrlich ascites and HeLa cervix uteri neoplastic cell lines, its mechanism of action is unknown. Others derivatives as desmethoxy-yatein and desoxypodophyllotoxin are responsible for cytotoxic and antitumoral activities [23, 24]. The analogue 4′-demethyl-epipodophyllotoxin inhibits DNA topoisomerase II [16, 24], and several series of nonlactonic podophyllic aldehyde analogues produce apoptosis induction of neoplastic cells without a previous tubulin inhibition [25].

In Mexico 28 species of* Linum* have been identified, of which 12 are endemic. The species of* Linum* are used in Mexican Traditional Medicine to treat respiratory infections, snake bites, malaria, leishmaniasis, gastrointestinal affections, inflammation, and cystitis as well as other conditions [26–29].

*Linum scabrellum* is a perennial herb endemic to Mexico that grows up to 40 or 50 cm, pubescent in its green parts with leaves alternated and flowering from June to December [28]. It is distributed in 7 states of the country: Querétaro, Guanajuato, Hidalgo, Oaxaca, Puebla, Nuevo León, and Tamaulipas. In a previous study, we reported the cytotoxic activity of three extracts (chloroform, butanol, and aqueous) prepared from aerial parts and roots of* L. scabrellum* against three human cancer cell lines. The obtained results showed that the most toxic activity was observed from the root's chloroformic extract against KB (epidermoid), HF6 (colon), and MCF7 (breast) carcinomas, exhibiting IC_50_ values that ranged from 0.2 to 4.8 *μ*g/mL [30].

The aim of the present investigation was to study the chemical composition of* Linum scabrellum* and to identify its cytotoxic constituents. This is the first phytochemical study that reports the complete metabolic content of this species that allowed the identification of 6MPTOX as the active metabolite against human cancer cell lines. In order to explore the mechanism of action of this compound, several studies were conducted to determine its effects on the cell cycle progression of PC3 prostate cancer cells, mitotic arrest, microtubule polymerization, binding affinity to tubulin, and cell death.

## 2. Materials and Methods

### 2.1. Plant Material

The wild plant* Linum scabrellum* was collected by M.S. Ivonne Alejandre García on November 2012 at 5 km to the south of Vizarrón, Cadereyta Queretaro, Mexico. A sample was authentified by BS Alejandro Flores and deposited at the HUMO Herbarium of the Universidad Autónoma del Estado de Morelos (UAEM), with the voucher number 27194.

### 2.2. Preparation of Extracts

The collected plant material was air dried and grounded using an electric mill (749 g), and four extracts of* L. scabrellum* were obtained with solvents of different polarity (hexane, ethyl acetate, dichloromethane, and methanol). The extracts of dichloromethane and methanol exhibited the major content of secondary metabolites; nonpolar metabolites were present in the dichloromethane extract while polar metabolites were in the methanol extract. The grounded plant was extracted with CH_2_Cl_2_:MeOH (50/50 v/v), in order to obtain the majority of the compounds, and sonicated during 30 min (×3). The extract was concentrated in a Buchi rotary evaporator at 40°C and stored at room temperature for further chemical fractionation. The extract yield was 19.4 g.

### 2.3. Isolation of Compounds

Compounds were isolated by means of open column chromatography (CC). The isolation procedures and purity of compounds were checked by thin layer chromatography (TLC), visualized by means of UV light, and sprayed with Ce(SO_4_)_2_·2(NH_4_)_2_SO_4_·2H_2_O. The UV spectra were recorded on a Hewlett Packard 8452A spectrophotometer. All ^1^H, ^13^C, and 2D NMR experiments were recorded in CDCl_3_, acetone-*d*_6_, and CD_3_OD on a Varian Unity 400 spectrometer at 400 MHz for ^1^H NMR, and at 100 MHz for ^13^C NMR. FABMS spectra were recorded on a JEOL JMX-AX 505 HA mass spectrometer. The IR spectrum was recorded on a Perkin Elmer FTIR spectrophotometer (Perkin Elmer, USA) using KBr, measured in cm^−1^. Melting points were determined on a Fisher-Johns Melting Point apparatus. Optical rotations were measured on a Perkin-Elmer 341 digital polarimeter at 25°C. All the reagents and solvents used were of analytical grade. The CH_2_Cl_2_:MeOH extract (19.4 g) was fractionated in an open chromatographic column (60 cm × 9 cm) previously packed with silica gel (582 g, 70–230 mesh; Merck) and eluted with a dichloromethane/methanol gradient system, starting with 100% of the less polar solvent and subsequently increasing the methanol. Seventy-eight fractions of 100 mL each were collected and concentrated by vacuum distillation and grouped according to their chemical content as monitored by TLC. Eight groups of fractions were obtained: F-1 (1.3 g, 100 : 0), F-2 (2.5 g, 98 : 2), F-3 (1.0 g, 96 : 4), F-4 (1.82 g, 94 : 6), F-5 (3.71 g, 92 : 8), F-6 (2.88 g, 90 : 10), F-7 (2.75 g, 85 : 15), and F-8 (0.90 g, 0 : 100). In the less polar fraction (F-1) oleic acid (**1**, 180 mg) and octadecenoic acid (**2**, 320 mg) were identified. After a successive chromatographic process using CH_2_Cl_2_:MeOH gradient system, F-2 (100 : 00→97 : 03) yielded stigmasterol (**3**, 25 mg) and *α*-amyrin (**4**, 14 mg), F-5 (100 : 00→85 : 15) afforded pinoresinol (**5**, 4.9 mg), 6-methoxypodophyllotoxin (**6**, 40 mg), and coniferin (**7**, 6.5 mg) and F-6 (100 : 00→80 : 20) yielded 6-methoxypodophyllotoxin-7-O-*β*-D-glucopyranoside (**8**, 503.1 mg).

### 2.4. Cytotoxic Assay

The CH_2_Cl_2_:MeOH of aerial and root extracts as well as compounds** 6**,** 7**, and** 8** were subjected to a cytotoxic evaluation using breast (MCF7), colon (HF6), prostate (PC3), and cervical (SiHa) human cancer cell lines from ATCC (American Type Culture Collection, USA), along with normal human fibroblast cells (HSF-30) at passage number 33 (In Vitro Co.). Cell cultures were grown in RPMI-1640 medium (Sigma Aldrich) supplemented with fetal bovine serum 10% (SFB, Invitrogen), NaHCO_3_ 7.5%, and cultivated in 96-well plates (10^4^ cells/mL) at 37°C with CO_2_ 5% (humidity 100%). Normal human fibroblasts were grown in DMEM medium (Invitrogen) supplemented with fetal bovine serum 10%. The cells in a log phase of their growth cycle were treated in triplicate with various concentrations of the test samples (0.16–20 *μ*g/mL) dissolved in dimethyl sulfoxide (DMSO, Sigma Aldrich) and incubated for 72 h in the conditions described above. In order to guarantee that the cells were in exponential growth, the criteria of confluence between 60–70% were adopted. The cell concentration was determined by the NCI sulforhodamine method [31]. The results were expressed as the dose that inhibits 50% control growth after the incubation period (IC_50_). The values were estimated from a log⁡10 plot of the drug concentration against the percentage of viable cells. The standards included as controls were PTOX and Taxol (Sigma Aldrich). Extracts with IC_50_ ≤ 20 *μ*g/mL and compounds with IC_50_ ≤ 4 *μ*g/mL were considered active according to the National Cancer Institute (NCI) guidelines [32]. The data analysis was processed using the program ORIGIN 8.0, IC_50_ values were obtained by a regression curve with coefficient factors *R* ^2^ between 0.80 and 0.99.

### 2.5. Cell Cycle Analysis

Prostate cancer cells PC3 (1 × 10^5^) were plated in 6-well plates and allowed to attach overnight at 37°C in 5% CO_2_. Exponential growing cells were exposed at four different concentrations of 6MPTOX in accordance with IC_50_ values (0.011 *μ*M, 0.005 *μ*M, 0.002 *μ*M, and 0.001 *μ*M) for 72 h. Cells from each treatment were trypsinized and collected into single cell suspensions, centrifuged, and fixed in cold ethanol (70%) overnight at −20°C. The cells were then treated with RNase (0.01 M, Sigma Aldrich) and stained with propidium iodide (PI) (7.5 *μ*g/mL, Invitrogen) for 30 min in the dark, PI has the ability to bind to DNA molecules, and then RNase was added in order to allow PI to bind directly to DNA. The percentage of cells in G1, S, and G2 phases was analyzed with a flow cytometer (Becton, Dickinson, FACS Calibur, San Jose, CA); the number of cells analyzed for each sample was 10,000. Data obtained from the flow cytometer were analyzed using the FlowJo Software (Tree Star, Inc., Ashland, OR, USA) to generate DNA content frequency histograms, and to quantify the number of cells in the individual cell cycle phases.

### 2.6. Immunofluorescence of Histone H3 Phosphorylated

PC3 cells were grown in RPMI medium, and 7.5 × 10^4^ cells were added in 24-well culture plates containing slides and allowed to attach overnight at 37°C in 5% CO_2_. Then, they were treated with 6MPTOX (0.005 *μ*M), PTOX (0.002 *μ*M), and DMSO (2.25 *μ*M) at 37°C for 72 h. The cells were fixed with PFA (*p*-formaldehyde) 4% in PEM buffer PIPES (0.1 M), EGTA (2 mM), and MgSO_4_ (1 mM), pH 6.95. After 15 min PFA/NaHCO_3_ was added and incubated for 45 min at room temperature. The slides were rinsed with PBS, treated with 0.1% Triton X-100 (Sigma Aldrich), and then incubated with the primary antibody antiphospho-histone H3 (1 : 500, Santa Cruz Biotechnology) overnight at 4°C. A secondary antibody anti-rabbit Alexa 488 (1 : 1000, Molecular Probes) was added and incubated for 1 h at 37°C. The cells were stained with 0.4 *μ*g/mL 4,6-diamidino-2-phenylindole (DAPI, Molecular Probes, Eugene, OR) in PBS for 10 min, then mounted, and imaged by fluorescence microscopy.

### 2.7. Immunofluorescence of *α*-Tubulin

PC3 cells were grown in RPMI medium, and 7.5 × 10^4^ cells were added in 24-well culture plates containing slides and allowed to attach overnight at 37°C in 5% CO_2_. Then they were treated with 6MPTOX (0.005 *μ*M), PTOX (0.002 *μ*M), and DMSO (2.25 *μ*M-0.02%) at 37°C for 72 h. The cells were fixed with PFA (paraformaldehyde) 4% in PEM (buffer). After 15 min PFA/NaHCO_3_ was added and incubated for 45 min at room temperature. The slides were rinsed with PBS and treated with 0.1% Triton X-100 (Sigma Aldrich) and then incubated with the first antibody anti-*α*-tubulin (1 : 300, Sigma Aldrich) overnight at 4°C. A second antibody anti-mouse Alexa 647 (1 : 1000, Molecular Probes) was added and incubated for two hours at 37°C. The cells were stained with Sytox Green (1 : 5000) for one hour, mounted, and imaged by confocal microscopy.

### 2.8. Determination of the Binding Site and Affinity Constant *K*_*b*_

The binding affinity constant (*K*_*b*_) of 6MPTOX was determined following the method described previously [33]. Displacement of the colchicine analogue MTC [2-methoxy-5-(2,3,4-trimethoxyphenyl)-2,4,6-cycloheptatrien-1-one], a commercial reversible tubulin ligand in the colchicine site that binds to the heterodimer *α*/*β* tubulin, was evaluated. Initially, mixtures of tubulin and MTC (5 *μ*M) were incubated in 10 mM of buffer GAB (NaPi with 0.1 mM GTP and pH 7). Subsequently, increasing concentrations of the above tested compound and controls were added and incubated for 10 min between each concentration at 25°C in the fluorometer Horiba Fluoromax-2, with *λ* exc = 350 nm and *λ* em = 423 nm. Binding constants were calculated by the decrease of the fluorescence of MTC due to competition for the same site. Data processing was analyzed using the Equigra v5 software [34].

### 2.9. Cell Death

PC3 cells (7.5 × 10^4^ cells/mL) were seeded in 6-well plates and incubated for 18 h. Exponentially growing cells were treated for 72 h with RPMI medium added with 0.02% of DMSO (control) and with four different concentrations of 6MPTOX (0.011 *μ*M, 0.005 *μ*M, 0.002 *μ*M, and 0.001 *μ*M) in accordance with the IC_50_ value of the compound. Cells treated with H_2_O_2_ were used as apoptotic death control [35] and for the necrotic control PC3 cells were treated with boiling water. Then, each cell culture was washed with 100 mL of PBS and stained with 100 *μ*L AO/EB solution (100 *μ*g/mL AO, 100 *μ*g/mL EB), according to reported procedures [36]. The cells were observed using a fluorescence microscope (Olympus Co., Tokyo, Japan, with emission at 521 nm). AO/EB are intercalating nucleic acid-specific fluorochromes, and when bounded to DNA they emit green and orange fluorescence, respectively. It is well known that AO can pass through cell membranes, but EB cannot. Under the fluorescence microscope, living cells appear green. Necrotic cells stain red but have a nuclear morphology resembling that of viable cells. Apoptotic cells appear green, and morphological changes such as formation of apoptotic bodies are observed. The criteria for identification are as follows: (i) viable cells appear to have green nucleus with intact structure; (ii) early apoptosis cells exhibit a bright-green nucleus showing condensation of chromatin; (iii) late apoptosis appears as dense orange areas of chromatin condensation; and (iv) orange intact nucleus depicts secondary necrosis [37].

## 3. Results and Discussion

### 3.1. Cytotoxic Activity and Chemical Composition of* Linum scabrellum*

**Figure 1:**
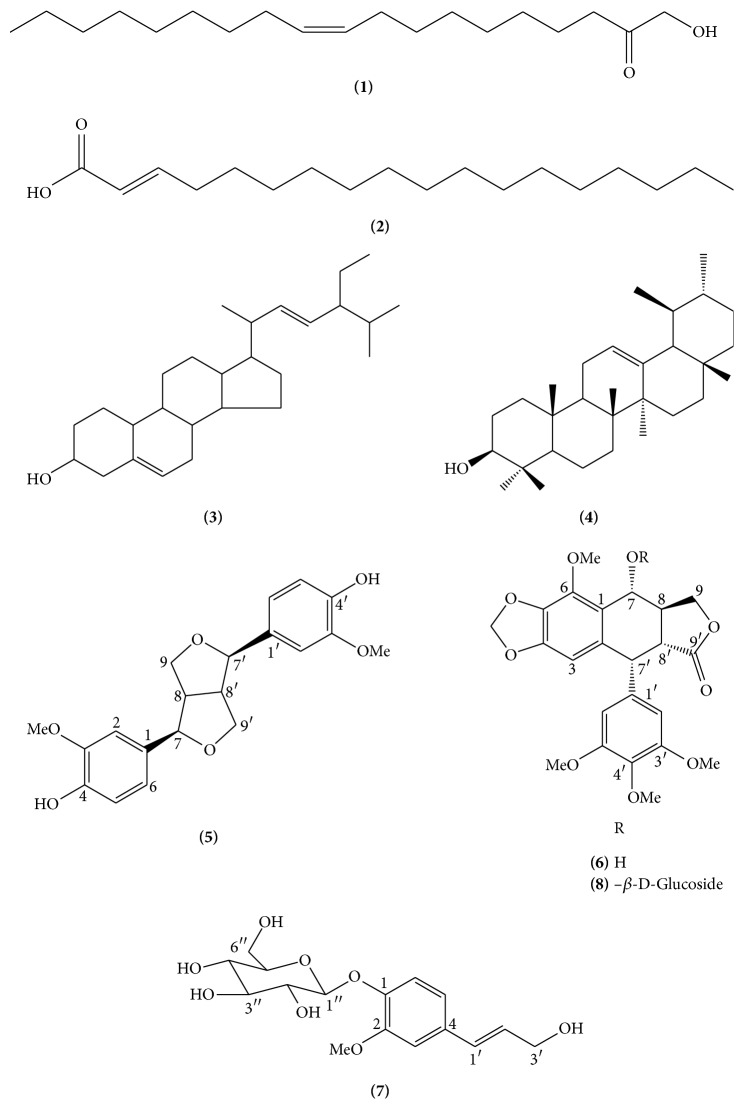
Chemical structure of* Linum scabrellum* secondary metabolites.

The CH_2_Cl_2_:MeOH extract from aerial parts was noncytotoxic to the tested cell lines (IC_50_ ≤ 20 *μ*g/mL). The CH_2_Cl_2_:MeOH roots extract of* L. scabrellum* presented cytotoxic activity against the four carcinoma cell lines exhibiting IC_50_ values from 0.56 to 1.54 *μ*g/mL as well as toward the normal cell line with an IC_50_ value of 1.02 *μ*g/mL (Table 1). The first complete phytochemical profile of* L. scabrellum* was established in order to identify the cytotoxic metabolites. In the CH_2_Cl_2_:MeOH extract, eight compounds were identified and characterized (Figure 1), three of them** (5**,** 6**, and** 8)** belong to the lignan group. Four compounds, oleic acid** (1)**, octadecenoic acid** (2)** stigmasterol** (3)**, and *α*-amyrin** (4)**, were identified by their spectroscopic data ^1^H and ^13^C and cochromatography with authentic samples available on our laboratory. The other four compounds were identified by their spectroscopic data ^1^H, ^13^C NMR, spectra mass, mp, UV, IR, and 2D experiments (COSY, HSQC) as follows: F-5 was subjected to column chromatography packed with silica gel (115 g) and eluted with a gradient system of CH_2_Cl_2_:MeOH (100 : 00→90 : 10) obtaining 46 fractions of 25 mL each and were grouped in six groups according to their similarity in thin layer chromatography (F-5A→ F-5F).

Fraction F-5B eluted with 98:02 CH_2_Cl_2_:MeOH afforded 4.9 mg of pinoresinol (**5**), isolated as amorphous powder, mp 121–22°C (lit: 122°C) an optically active powder [*α*]^24^_D_  −82.3° (c 0.012, Me_2_CO), and had the formula C_20_H_22_O_6_ derived from its positive FABMS [M+H]^+^ ion at* m/z* 359. IR (KBr, *υ*_max_ in cm^−1^): 3210, 2870, 1600, 1465 1050. UV *λ*_max_ (nm): 232, and 280. ^1^H NMR (400 MHz, CDCl_3_) *δ*_H_: 6.89 (*d*,* J* = 1.2 Hz; H-2, H-2′), 6.88 (*d*,* J* = 8.4 Hz; H-5, H-5′), 6.81 (*dd*,* J* = 2.1 and 8.4 Hz; H-6, H-6′), 4.73 (*d*,* J* = 4.2 Hz; H-7, H-7′), 3.09 (*c*, H-8, H-8′), 4.24 (*dd*,* J* = 7 and 9.1 Hz; 9-H*β*, 9′-H*β*), 3.87 (*dd*,* J* = 3.5 and 9.1 Hz; 9-H*α*, 9′-H*α*), 3.90 (*s*, C3′-OMe, C3-OMe). ^13^C NMR (100 MHz, CDCl_3_) *δ*_C_: 132.90 (C-1, C-1′), 108.57 (C-2, C-2′), 146.68 (C-3, C-3′), 145.22 (C-4, C-4′), 114.24 (C-5, C-5′), 118.96 (C-6, C-6′), 85.86 (C-7, C-7′), 54.16 (C-8, C-8′), 71.66 (C-9, C-9′). On the basis of the spectral data, compound** 5** was identified as (-)-pinoresinol [38].

One of the fractions F-5C eluted with 96:04 CH_2_Cl_2_:MeOH yielded 40 mg of compound** 6**, obtained as a white amorphous powder, mp 202-203°C (lit: 203°C). The positive FABMS spectrum showed the [M + H]^+^ ion peak at* m/z* 445 with molecular formula C_23_H_24_O_9._ IR (KBr, *υ*_max_ in cm^−1^): 3220, 1768, 1600. UV *λ*_max_ (nm): 272, and 282. ^1^H NMR (400 MHz, acetone-d_6_) *δ*_H_: 6.30 (s, H-3), 5.03 (*d*,* J* = 9.8 Hz, H-7), 2.88 (*m*, H-8), 4.06 (*dd*,* J* = 8.4 and 10 Hz; H-9*β*), 4.64 (*t*,* J* = 7.7 and 8.4 Hz; H-9*α*), 6.44 (*s*, H-2′, H-6′), 4.53 (*d*, *J* = 4.9 Hz, H-7′), 2.75 (*dd*, *J* = 4.2 and 14.7 Hz; H-8′), 3.78 (*s*, C3′-OMe, C5′-OMe), 3.81 (*s*, C4′-OMe), 3.78 (*s*, C6-OMe), 5.95 (*s*, –O-CH_2_-O-). ^13^C NMR (100 MHz, acetone-d_6_) *δ*_C_: 124.93 (C-1), 137.04 (C-2), 104.36 (C-3), 149.45 (C-4), 132.84 (C-5), 141.57 (C-6), 70.52 (C-7), 39.01 (C-8), 71.92 (C-9), 134.71 (C-1′), 108.09 (C-2′), 152.61 (C-3′), 134.93 (C-4′), 152.61 C-5′), 108.09 (C-6′), 45.11 (C-7′), 44.53 (C-8′), 174.44 (C-9′), 56.18 (C3′-OMe), 60.77 (C4′-OMe), 56.18 (C5′-OMe), 59.93 (C6-OMe), 101.37 (-O-CH2-O-). On the basis of the spectral data, compound** 6** was identified as 6-methoxypodophyllotoxin [39].

Fraction F-5C eluted with 90 : 10 CH_2_Cl_2_:MeOH yielded 6.5 mg of coniferin (**7**), obtained as an amorphous solid, mp 180–82°C [lit: 182°C]. Positive FABMS* m/z* 342 [M + H]^+^, with molecular formula C_16_H_22_O_8_. IR (KBr, *υ*_max_ in cm^−1^): 3020, 1060, 940. UV *λ*_max_ (nm): 256 and 292. [*α*]^24^_D_  −66.3 (c 0.010, Me_2_CO). ^1^H NMR (400 MHz, CDCl_3_) *δ*_H_: 7.07 (*d*,* J*=2.4 Hz; H-3), 6.89 (*dd*,* J* = 1.6 and 8 Hz; H-5), 7.09 (*d*,* J* = 8 Hz; H-6), 6.52 (*d*,* J* = 15.2 Hz; H-1′), 6.29 (*td*,* J* = 5.6 and 16 Hz; H-2′), 4.19 (*t*,* J* = 5.6 Hz; H-3′), 4.87 (*d*,* J* = 7.7 Hz; H-1′′), 4.23 (*t*,* J* = 7.8 Hz, H-2′′), 4.22 (*t*,* J* = 7.6 Hz, H-3′′), 4.21 (*dd*,* J* = 7.6 and 7.8 Hz, H-4′′), 4.05 (*m*, H-5′′), 3.54 (*t*,* J* = 9.6 Hz, H-6′′a), 3.70 (*dd*,* J* = 4.5 and 9.8 Hz, H-6′′b), 3.79 (*s*, C2-OMe). ^13^C NMR (100 MHz, acetone-d_6_) *δ*_C_: 150.01 (C-1), 146.54 (C-2), 110.24 (C-3), 132.36 (C-4), 117.11 (C-5), 119.23 (C-6), 128.88 (C-1′), 128.87 (C-2′), 62.36 (C-3′), 55.53 (C2-OMe), 101.69 (C-1′′), 73.92 (C-2′′), 77.07 (C-3′′), 70.69 (C-4′′), 76.93 (C-5′′), 61.82 (C-6′′). On the basis of spectral data, compound** 7** was identified as phenylpropane glycoside coniferin (**7**) [40].

From the fraction F-6, compound** 8** was isolated as an amorphous yellow solid, mp 287°C [lit: 287°C]. The positive FABMS spectrum showed the [M + H]^+^ ion peak at* m/z* 607, with molecular formula C_29_H_34_O_14._ IR (KBr, *υ*_max_ in cm^−1^): 3020, 1746, 1620, 1050, 920. UV *λ*_max_ (nm): 276 and 284. ^1^H NMR (400 MHz, CD_3_OD) *δ*_H_: 6.28 (*s*, H-1), 5.48 (*d*, 7.2 Hz; H-7), 2.90 (*m*, H-8), 4.23 (*t*,* J* = 8.8 and 9.6 Hz; H-9*β*), 4.71 (*t*,* J* = 8.4 and 8.6 Hz; H-9*α*), 6.31 (*s*, H-2′, H-6′), 4.51 (*d*,* J* = 4.8 Hz; H-7′), 3.25 (*dd*,* J* = 4.8 and 14 Hz; H-8′), 3.72 (*s*, C3′-OMe, C5′-OMe), 3.99 (*s*, C4′-OMe), 3.68 (*s*, C6-OMe), 6.02 (*s*,-O-CH2-O-), 4.54 (*d*,* J* = 7.6 Hz; H-1′′), 3.28 (*t*,* J* = 9.2 Hz; H-2′′), 3.35 (*t*,* J*= 7.2 Hz; H-3′′), 3.41 (*dd*,* J* = 7.2 y 6.8 Hz; H-4′′), 3.43 (*m*, H-5′′), 3.76 (*dd*,* J* = 2.2 and 10 Hz, H-6′′a), 3.84 (*dd*,* J* = 5.4 and 10.2 Hz, H-6′′b). ^13^C NMR (100 MHz, CD_3_OD) *δ*_C_: 124.26 (C-1), 136.28 (C-2), 105.74 (C-3), 149.68 (C-4), 135.89 (C-5), 140.26 (C-6), 72.78 (C-7), 39.87 (C-8), 72.39 (C-9), 136.73 (C-1′), 108.58 (C-2′), 152.89 (C-3′), 136.54 (C-4′), 152.89 (C-5′), 108.58 (C-6′), 45.86 (C-7′), 46.23 (C-8′), 176.67 (C-9′), 56.24 (C3′-OMe, C5′-OMe), 60.76 (C4′-OMe), 60.76 (C6-OMe), 102.34 (-O-CH2-O-), 100.46 (C-1′′), 74.87 (C-2′′), 77.85 (C-3′′), 71.67 (C-4′′), 78.13 (C-5′′), 63.08 (C-6′′). On the basis of spectral data, compound** 8** was identified as 6-methoxypodophyllotoxin 7-*O*-*β*-D-glucopyranoside [40].

The obtained data were compared with the literature [41–43] (see Supplementary Material Figures 1–6 in Supplementary Material available online at http://dx.doi.org/10.1155/2015/298463).

Some studies reported that compounds** 1**,** 2**, and** 4** possess anti-inflammatory activity [42] while compound** 5** has chemopreventive properties. [38]. Even though all of the 8 compounds had been previously reported in members of the genus* Linum* [44–47], this is the first time that they were identified in* Linum scabrellum.*

### 3.2. Cytotoxic Activity of Purified Compounds

The isolated compounds** 6**,** 7**, and** 8** were evaluated for cytotoxic effect against the above mentioned cell lines. Compounds** 7** and** 8** were nonactive (IC_50_ ≥ 20 *μ*g/mL), while compound** 6** (6MPTOX) showed an important cytotoxic activity with IC_50_ values ranging from 0.0632 to 2.7433 *μ*g/mL against the cancer tested cell lines (Table 1). It is important to point out that 6MPTOX was also toxic against the normal fibroblasts. These data showed that the inhibitory effect of 6MPTOX as well as that of the CH_2_Cl_2_:MeOH roots extract of* Linum scabrellum* is not specific against certain carcinomas but rather exerts a general cell toxic action. Because the important cytotoxic activity demonstrated by 6MPTOX, we decided to investigate the mechanism of action of this compound.

### 3.3. Effect of 6MPTOX on PC3 Cell Cycle Arrest

**Figure 2:**
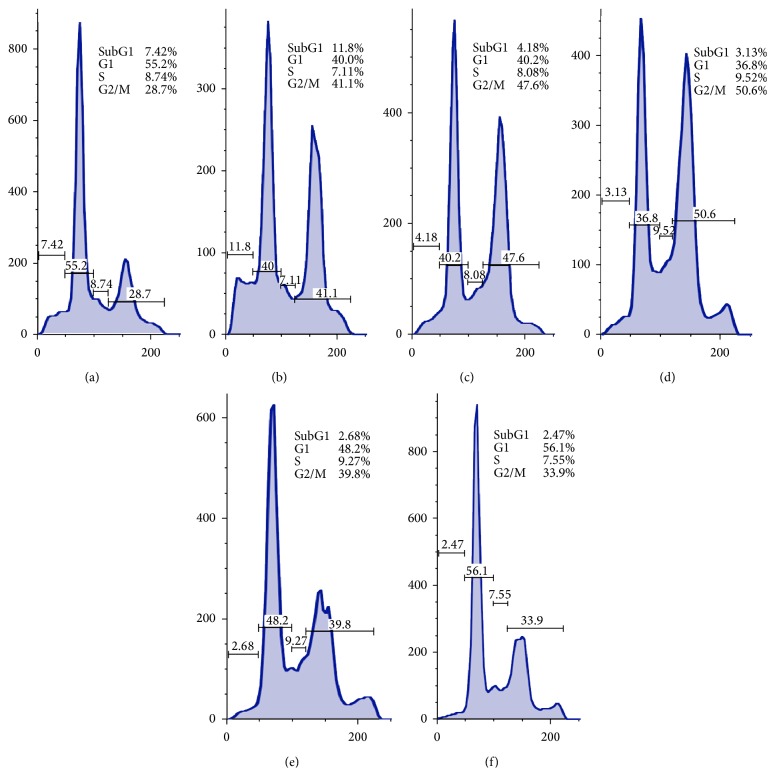
Effect of 6MPTOX on cell cycle in the prostate PC3 cell line. (a) Negative control with DMSO 0.02%; (b) PC3 cells exposed to 0.002 *μ*M PTOX as positive control; (c–f) PC3 cells exposed to different concentrations of 6MPTOX; (c) 0.011 *μ*M; (d) 0.005 *μ*M; (e) 0.002 *μ*M; (f) 0.001 *μ*M.

The effect of 6MPTOX in the cell cycle progression of PC3 cells was determined at four concentrations: 0.011 *μ*M, 0.005 *μ*M, 0.002 *μ*M, and 0.001 *μ*M. DMSO (2.55 *μ*M-0.02%) and PTOX (0.0024 *μ*M) were used as negative and positive controls, respectively.  Figure 2 displays DNA histograms of PC3 cell cycle. The 6MPTOX induced in PC3 a G2/M phase cell arrest. The effect was dose-dependent since cell arrest in G2/M was declining, while the G1 phase cell population was increasing when the 6MPTOX concentration decreased. 6MPTOX and PTOX at 0.0002 *μ*M induced a similar effect on G2/M cell arrest with 39.8% and 41.1% of G2/M cell population, respectively. These results suggest that 6MPTOX efficiently arrest cells at G2/M phase.

### 3.4. Effect of 6MOTX on Mitotic Arrest

**Figure 3:**
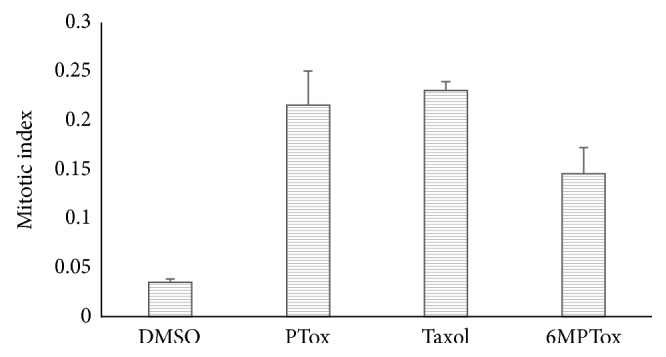
Effect of 6MPTOX on mitosis of the prostate cancer cell line PC3.

Histone H3 phosphorylated at serine 10 has long been used to identify mitotic cells nuclei in culture cell lines [48]. The proportion of cells exhibiting mitosis in the PC3 cultures was determined using an antibody antiphospho-histone H3, after treatment with 6MPTOX. The mitotic index was calculated by image analysis. Taxol and PTOX were used as positive controls, while DMSO was used as the negative control. 6MPTOX showed a mitotic index of 0.1, Taxol of 0.25, and PTOX of 0.2, while the negative control displayed a mitotic index of 0.035 (Figure 3). These results suggest that 6MPTOX induces mitotic arrest as did PTOX and Taxol, in contrast to the negative control. Inhibition of tubulin has been implicated in G2/M phase of the cell cycle arrest in various cancer cell lines [49]. It is well known that Taxol stabilizes microtubules protecting them from depolymerization, which blocks cells in mitosis and induces apoptosis [50], in contrast with PTOX, which promotes depolymerization by destabilizing microtubules and arrests the cell cycle in mitosis [51].

Our results showed that 6MPTOX induced a mitotic cell arrest by the observed high mitotic index and the G2/M cell arrest, suggesting a similar effect as that displayed by PTOX or Taxol. In order to determine the mechanism of action of 6MPTOX, we analyzed the effect of microtubule polymerization by 6MPTOX through disruption of *α*/*β* tubulin binding.

### 3.5. Effect of 6MPTOX on the *α*-Tubulin Polymerization

**Figure 4:**
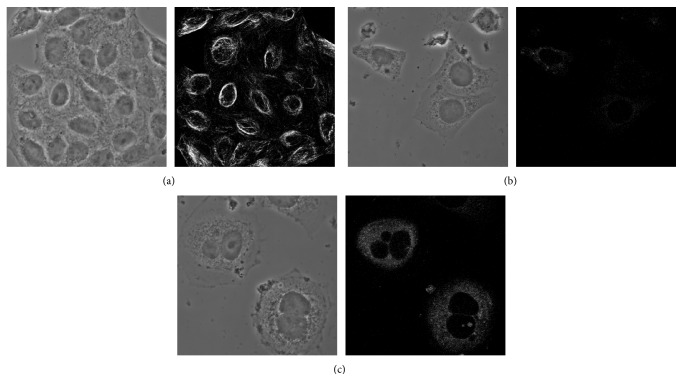
Effect of microtubules polymerization. (a) DMSO 0.02%, (b) 6MPTOX, and (c) PTOX.

Microtubules are in dynamic equilibrium with tubulin dimers as tubulin is polymerized into microtubules and depolymerized as free tubulin. This dynamic equilibrium is targeted by microtubule disrupting agents [52], which inhibit the organization of the mitotic spindle and arrest chromosomes in metaphase of mitosis [53]. The effect of 6MPTOX on the microtubule polymerization by immunofluorescence of *α*-tubulin was observed in PC3 cells by confocal microscopy.  Figure 4(b) shows that treatment with 6MPTOX inhibits the polymerization of microtubules possibly through binding to tubulin, of which effect was also observed by treatment with PTOX (Figure 4(c)). In the negative control (DMSO 0.02%, Figure 4(a)) no effect was appreciated.

These results suggest that 6MPTOX as well as PTOX act as microtubule-destabilizing agents, while cells treated with DMSO (0.02%) demonstrated a normal and intact tubulin organization (Figure 4(a)).

### 3.6. Determination of the Binding Site and Binding Constant (*K*_*b*_) of 6MPTOX

**Figure 5:**
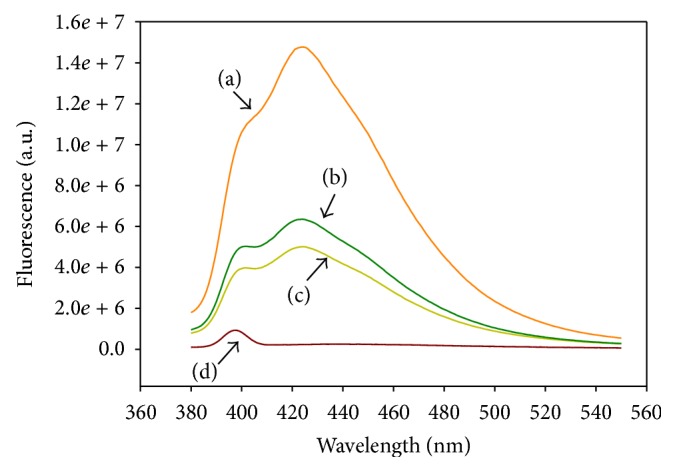
Displacement of MTC by 6MPTOX measured with fluorescence, (a) MTC with tubulin, (b) 6MPTOX, (c) PTOX, and (d) MTC.

To corroborate whether 6MPTOX binds to *α*/*β*-tubulin as does PTOX, 6MPTOX was assayed for its ability to displace MTC from its binding at the colchicine site of *α*/*β*-tubulin [54, 55].  Figure 5 shows fluorescence changes produced by the displacement of MTC binding to tubulin by both 6MPTOX and PTOX. The fluorescence spectra of MTC decreased by 43% with 6MPTOX indicating that 6MPTOX binds to the colchicine binding site, while PTOX decreased 65%. The binding constant for 6MPTOX was determined as *K*_*b*_ = 7.26 × 10^6^ M^−1^ by displacement of MTC in the colchicine binding site. MTC has *K*_*b*_ = 4.7 × 10^5^ M^−1^ [33], which indicated that 6MPTOX is a stronger inhibitor when compared to MTC.

### 3.7. Effect of 6MPTOX on Cellular Death of PC3 Cells

**Figure 6:**
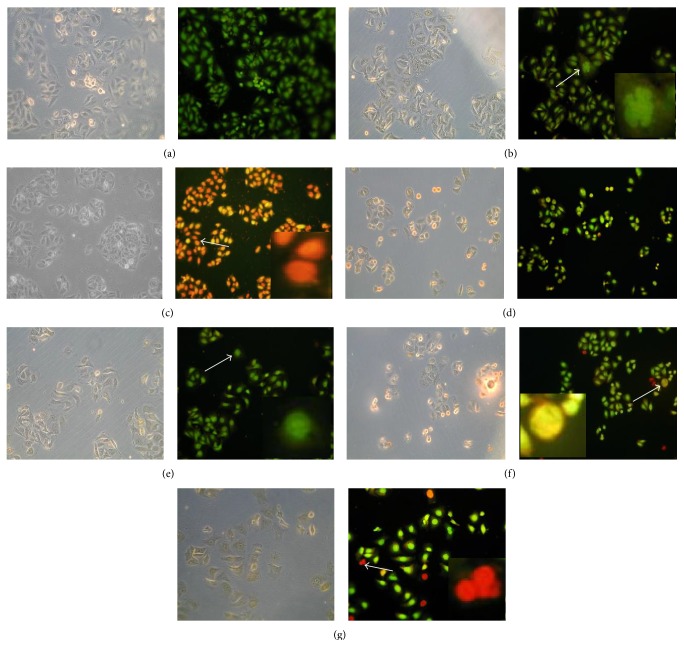
Effect of 6MPTOX on the prostate cancer cells (PC3). (a) Negative control; (b) apoptosis control; (c) necrosis control; (d–g) cells exposed to different concentrations of 6MPTOX; (d) 0.001 *μ*M; (e) 0.002 *μ*M; (f) 0.005 *μ*M; (6) 0.011 *μ*M. The arrow points out the cells that were amplified (40x). (b) Apoptotic cells; (c) necrotic cells; (e) apoptotic cells (0.002 *μ*M); (f) late apoptosis (0.005 *μ*M); (g) necrotic cells (0.011 *μ*M).

Cell death occurs through at least three morphologically distinct subroutines that have been named apoptosis, autophagy cell death (ACD), and necrosis [56]. Apoptosis is morphologically defined by nuclear shrinkage and fragmentation [57], whereas necrosis is defined by early permeabilization of the plasma membrane [58]. The effect of 6MPTOX on cellular death of prostate cancer cells was determined using four different concentrations. The compound 6MPTOX at a lower concentration of 0.001 *μ*M did not show any effect on cell death. When the concentration was increased to 0.002 *μ*M some apoptotic bodies were observed (Figure 6(e)); with 0.005 *μ*M, few cells were in late apoptosis and others were necrotic (orange or red color, Figure 6(f)); and at the highest concentration of 0.011 *μ*M the effect caused cell death mainly by necrosis (Figure 6(g)). Three controls were used: boiling water for necrosis, where most of the cells stained in red (Figure 6(c)); H_2_O_2_ for apoptosis, where some of the cells stained in green or orange with apoptotic bodies (Figure 6(b)), and a negative control with most of the cells stained in green (cells without treatment, Figure 6(a)). The effect of 6MPTOX on prostate cancer cells was dose-response, inducing apoptosis followed by necrosis when the concentration increased.

## 4. Conclusion

For the first time, the metabolic content of the Mexican species* L. scabrellum* was established. Eight compounds including lignans, terpenes, and fatty acids were isolated and identified. This study demonstrated the cytotoxic activity of 6MPTOX toward four selected human carcinomas and against a normal fibroblast cell line. Biochemical and biological experiments showed that 6MPTOX specifically arrested PC3 cells in their G2/M phase as well as a higher mitotic index. Moreover, at its lowest concentration it induces apoptosis as shown by an increase in its subG1 cell population and in stained apoptotic cells. Necrotic cell death was observed at higher concentrations. Data presented in this study show that 6MPTOX binds at the colchicine binding site of tubulin with a *K*_*b*_ = 7.26 × 10^6^ M^−1^, causing disruption of tubulin polymerization. These results showed that 6MPTOX inhibit tubulin polymerization and arrest cells in G2/M as a mechanism of cytotoxic activity in PC3 cells. This is the first report describing the mechanism of action of 6MPTOX.

## Supplementary Material

Supplementary Material provides all the spectroscopic data of isolated compounds (NMR and MS spectra).

## Figures and Tables

**Table 1:** IC_50_ values (*μ*g/mL) of dichloromethane/methanol extract and 6MPTOX isolated from *Linum scabrellum*.

Extract/compound	Cell lines				
	PC3	MCF7	HF6	SiHa	HFS-30
CH_2_Cl_2_:MeOH extract	1.60 ± 0.07	5.7 × 10^−1^ ± 0.07	5.7 × 10^−1^ ± 0.07	1.54 ± 0.07	1.02 ± 0.07
6MPTOX	1.7 × 10^−1^ ± 0.07	6.6 × 10^−2^ ± 0.07	7.9 × 10^−2^ ± 0.07	2.74 ± 0.07	6.0 × 10^−2^ ± 0.07
PTOX	3 × 10^−3^ ± 0.07	1 × 10^−4^ ± 0.07	1.4 × 10^−3^ ± 0.07	1.23 ± 0.07	1 × 10^−4^ ± 0.07
Taxol	6.2 × 10^−1^ ± 0.07	1.3 × 10^−1^ ± 0.07	2.8 × 10^−1^ ± 0.07	2.30 ± 0.07	1.2 × 10^−1^ ± 0.07

PC3: prostate cancer, MCF7: breast cancer, HF6: colon cancer, SiHa: cervical cancer, and HFS-30: fibroblasts.
