# Supplementary material for: Cytotoxic Activity and Chemical Composition of the Root Extract from the Mexican Species Linum scabrellum: Mechanism of Action of the Active Compound 6-Methoxypodophyllotoxin
Source: Evid Based Complement Alternat Med. 2015 Jul 12;2015:298463. doi: 10.1155/2015/298463 (PMC4515262; doi:10.1155/2015/298463)
Supplement: Supplementary file 1 — Supplementary Material provides all the spectroscopic data of isolated compounds (NMR and MS spectra). [file 298463.f1.pdf]

# Cytotoxic Activity and Chemical Composition of the Root Extracts from the Mexican Species *Linum scabrellum:* Mechanism of Action of the Active Compound 6-methoxypodophyllotoxin

Ivonne Alejandre-García[1], Laura Álvarez[2], Alexandre Cardoso-Taketa[1], Leticia González-Maya[3], Mayra Antúnez[2], Enrique Salas-Vidal[4], Fernando Díaz[5], Silvia Marquina-Bahena[2*], and María Luisa Villarreal[1]*

[1] Centro de Investigación en Biotecnología. Universidad Autónoma del Estado de Morelos.
[2] Centro de Investigaciones Químicas. Universidad Autónoma del Estado de Morelos.
[3] Facultad de Farmacia. Universidad Autónoma del Estado de Morelos.
[4] Instituto de Biotecnología. Universidad Nacional Autónoma México.
[5] Centro de Investigaciones Biológicas. Madrid, Spain.

# Supplementary Material

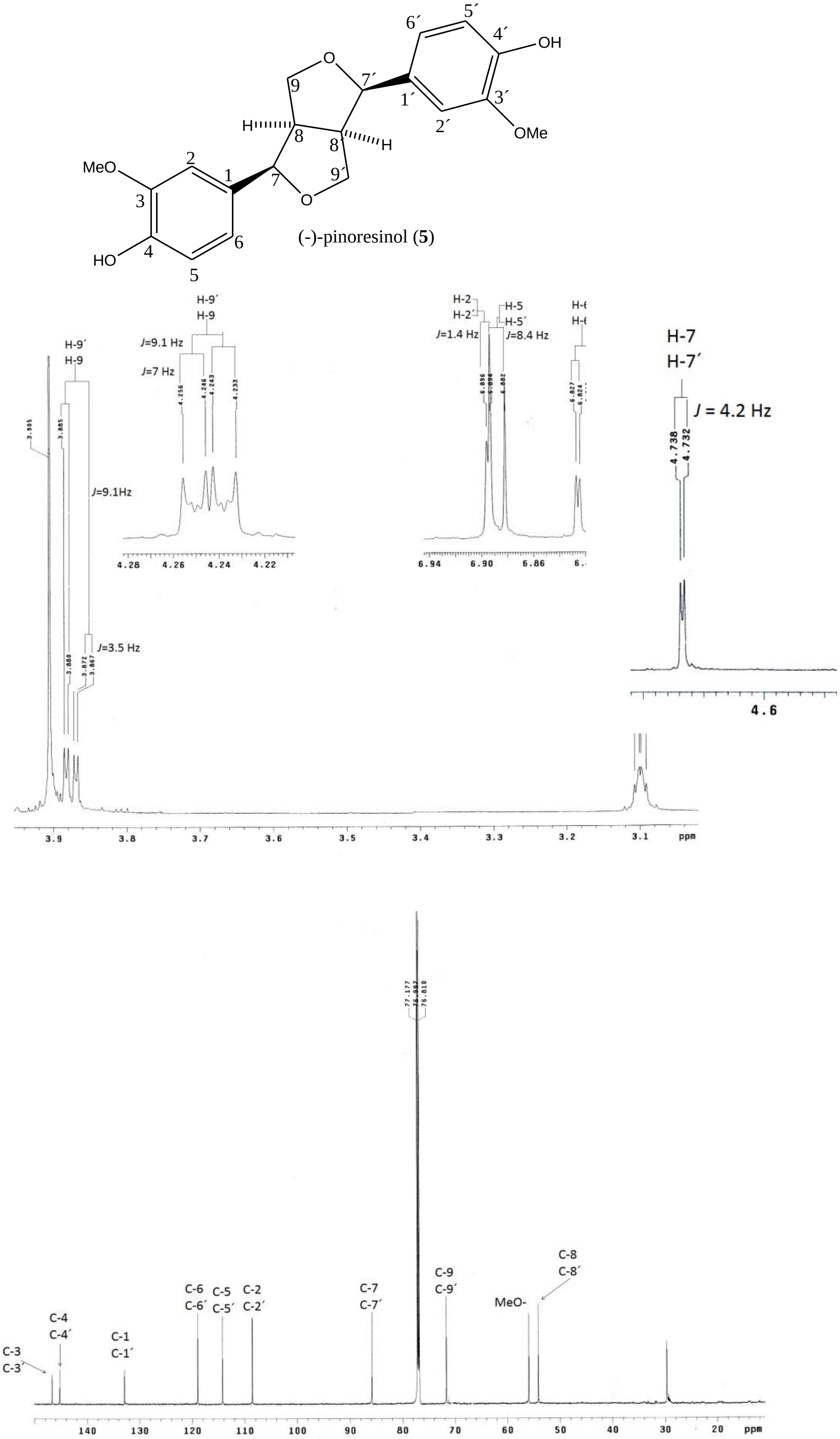


Figure 1. $^{1}H$ NMR and $^{13}C$ NMR spectra (400 and 100 MHz, $CDCl_3$) of (-)- pinoresinol (**5**)

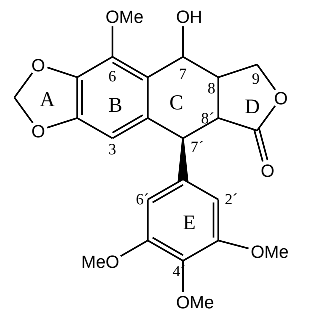


6-methoxypodophyllotoxin (**6**)

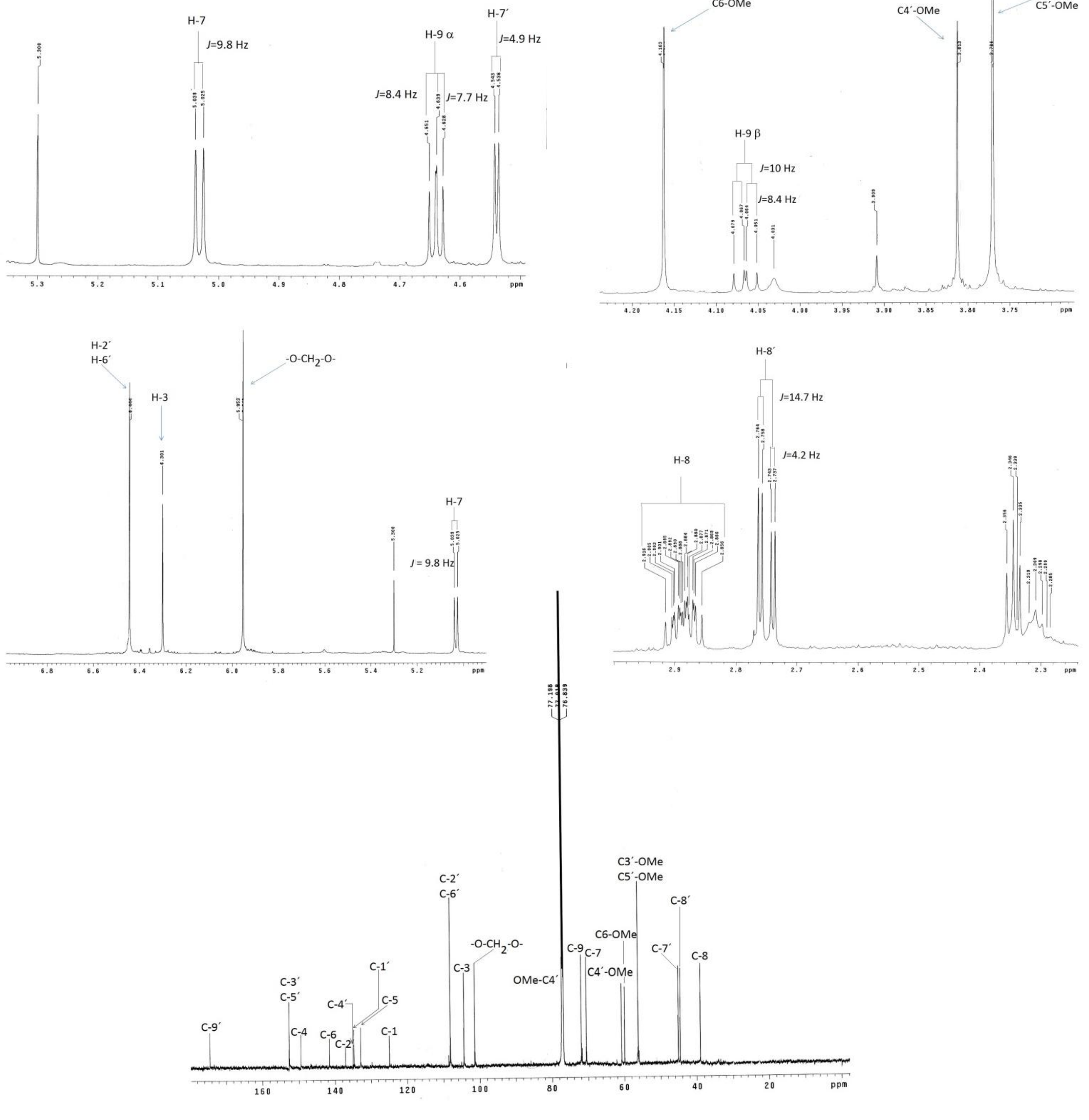


Figure 2. $^1$H NMR and $^{13}$C NMR spectra (400 and 100 MHz, $CDCl_3$) of 6-methoxypodophyllotoxin (**6**)

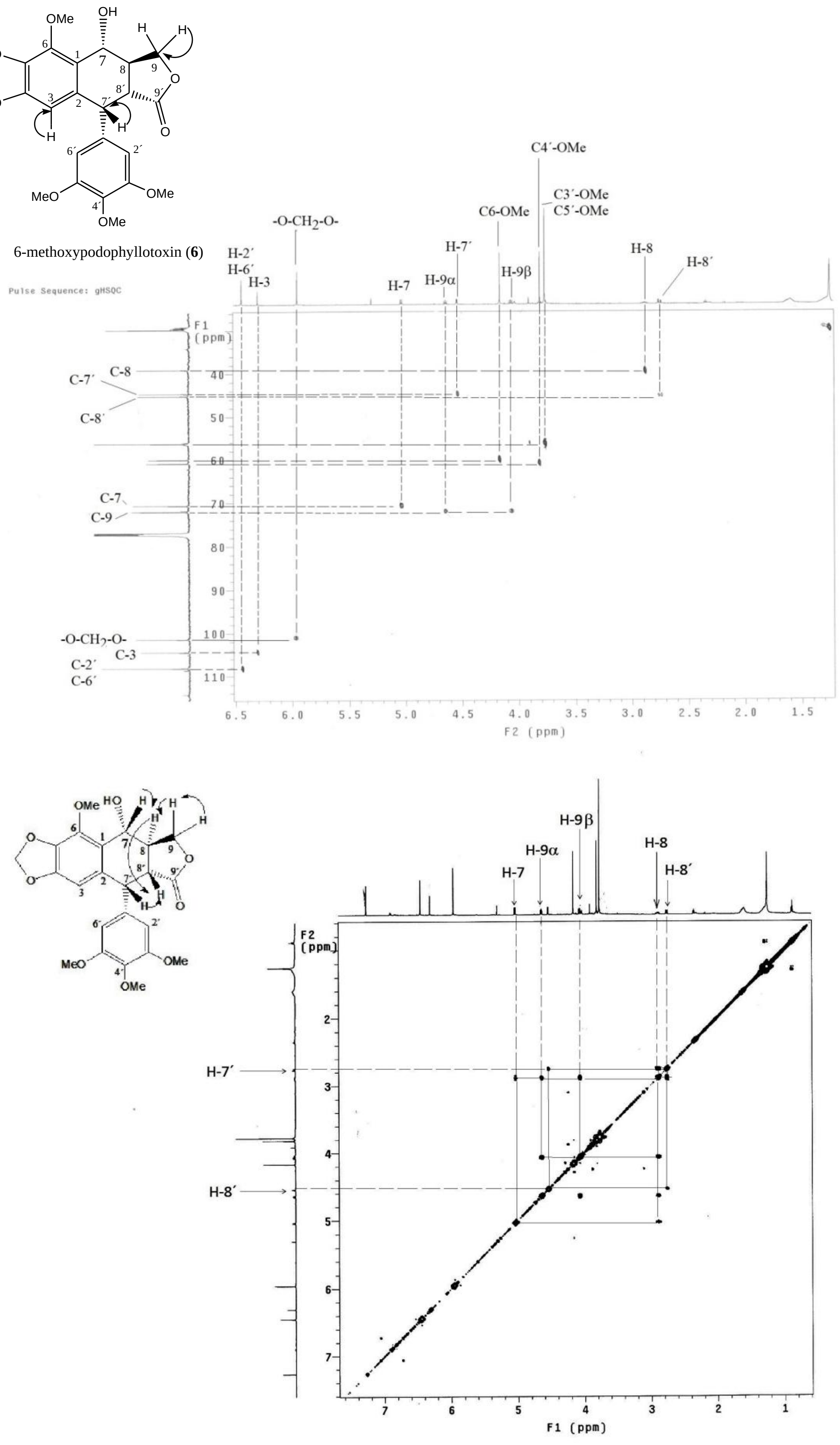


Figure 3 . HSQC and COSY spectra (400 MHz, CDCl3) of 6-methoxypodophyllotoxin (**6**)

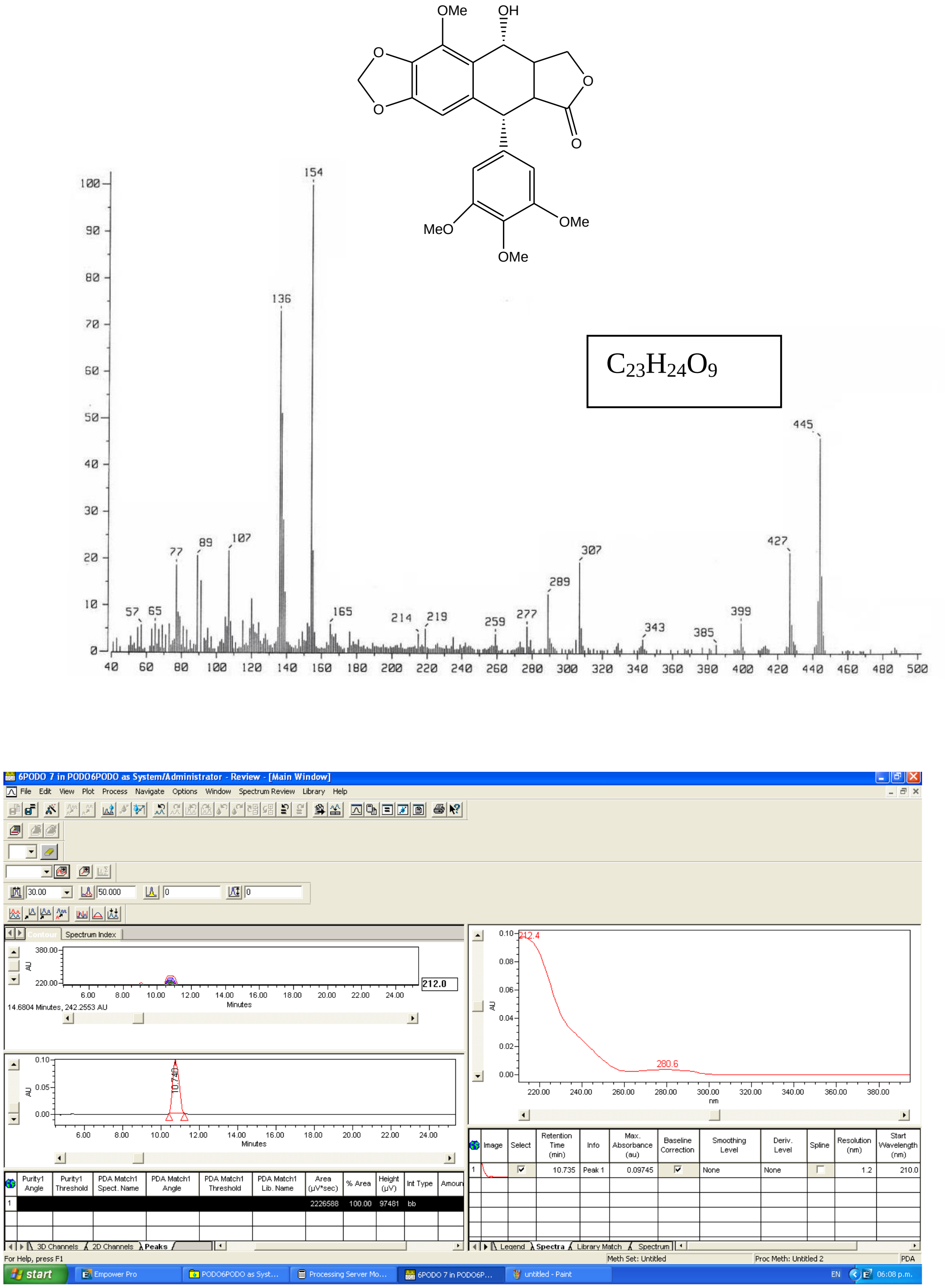


Figure 4. Positive FABMS and HPLC of 6-methoxypodophyllotoxin (**6**). 6-methoxypodophyllotoxin (**6**) was analyzed by high performance liquid chromatography, using a Waters 600 pump and Waters 2996 photodiode array detector. The analytical analysis was carried out with an isocratic solvent system (60% $CH_3CN$, 40% $H_2O$ with 0.0125% TFA) through a Shiseido Capcell pak C18 column (4.6 mm I.D. x 250 mm, 5 µm), and a flow rate of 1 mL/min. An injection volume of 20 µL, three injections were performed for each sample.

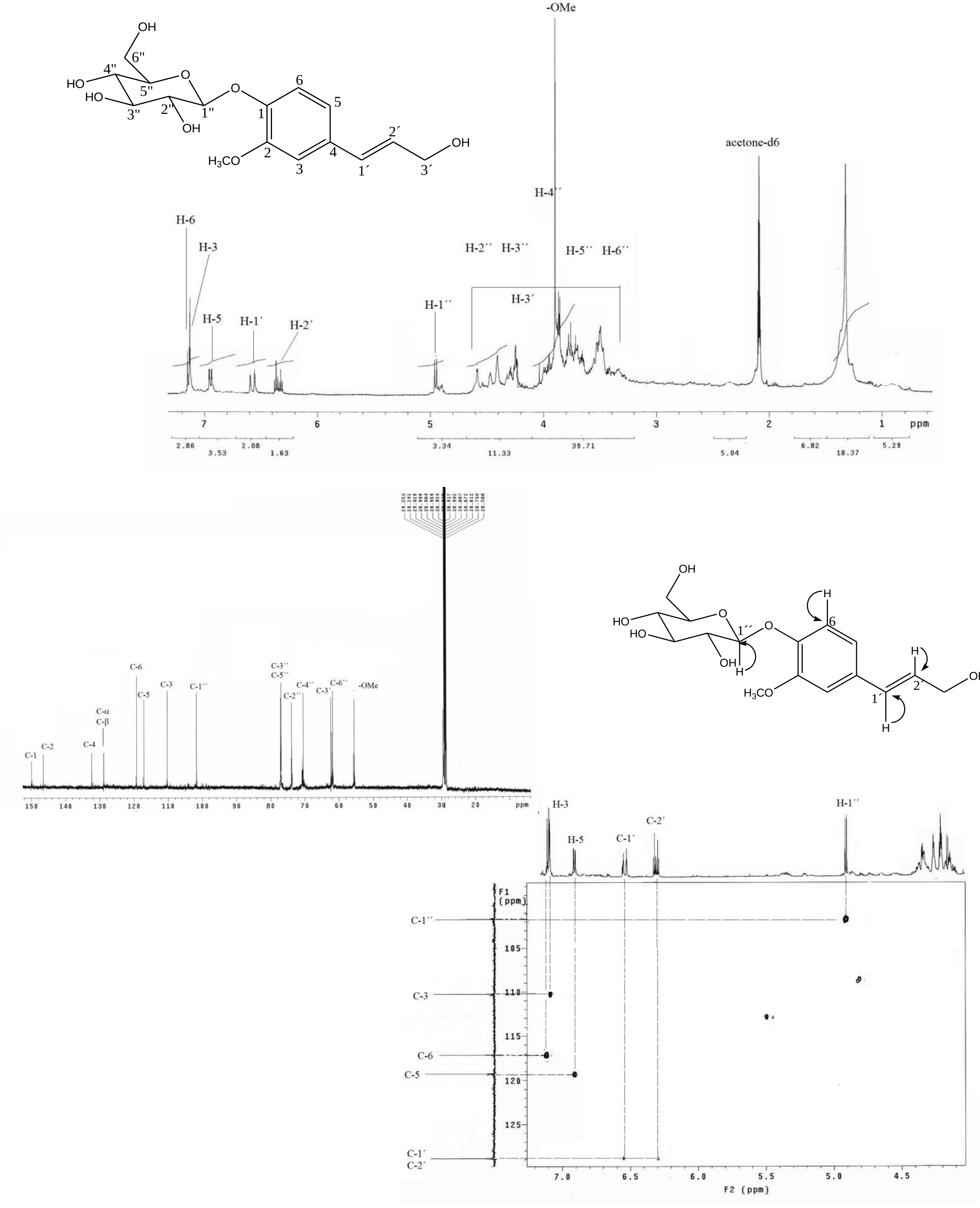


Figure 5. $^{1}$H NMR, $^{13}$C and HSQC NMR spectra (400 and 100 MHz, acetone-$d_6$) of coniferin (**7**)

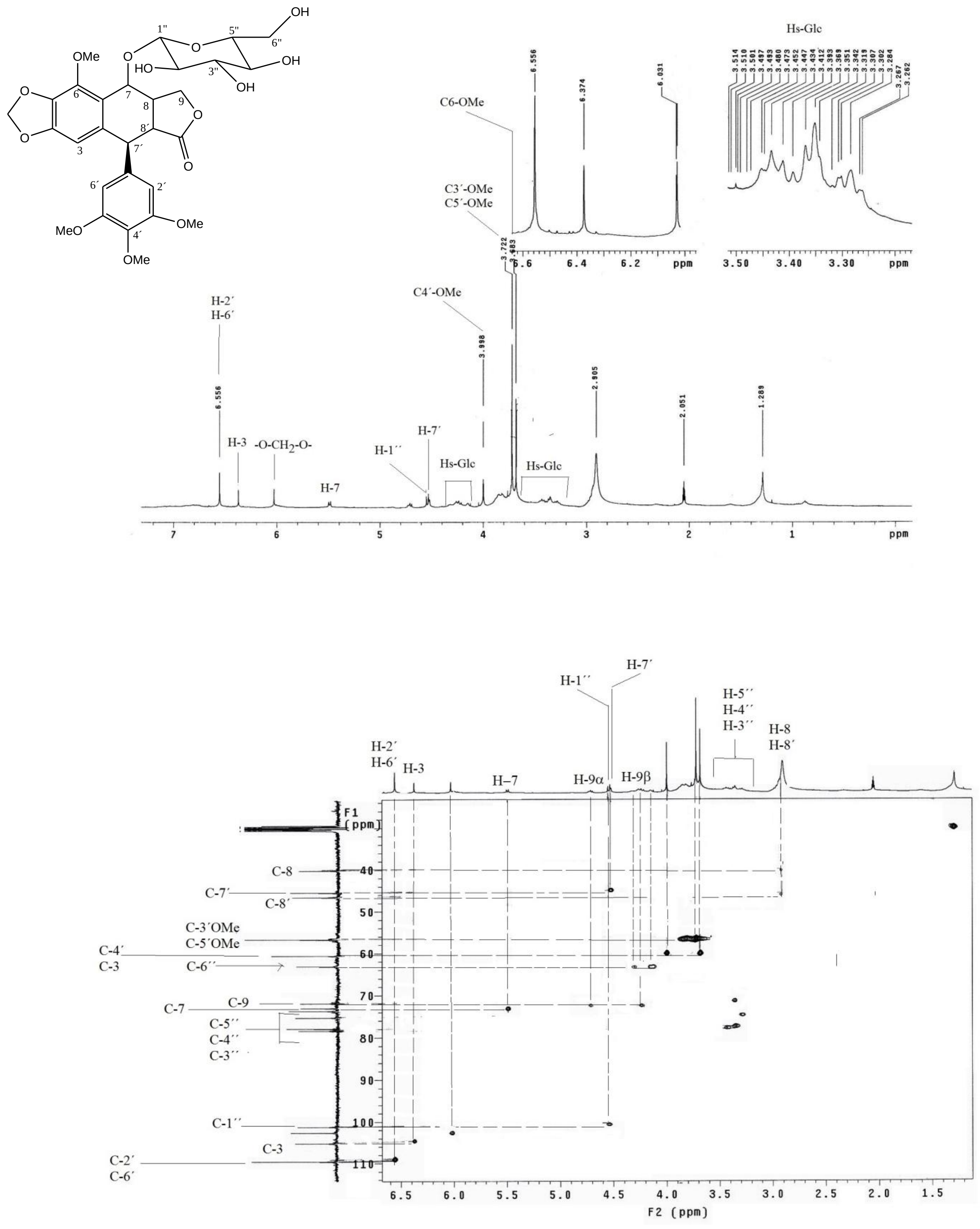


Figure 6. $^{1}H$ NMR and HSQC NMR spectra (400 and 100 MHz, acetone-d6) of 6-methoxypodophyllotoxin 7-*O*-β-D-glucopyranoside (**8**)
